# A psychometric assessment of Disturbances in Self-Organization symptom indicators for ICD-11 Complex PTSD using the International Trauma Questionnaire

**DOI:** 10.1080/20008198.2017.1419749

**Published:** 2018-01-17

**Authors:** Mark Shevlin, Philip Hyland, Neil P. Roberts, Jonathan I. Bisson, Chris R Brewin, Marylene Cloitre

**Affiliations:** ^a^ School of Psychology, Ulster University, Derry, Northern Ireland; ^b^ School of Business, National College of Ireland, Dublin, Ireland; ^c^ Centre for Global Health, Trinity College Dublin, Dublin, Ireland; ^d^ Psychology and Counselling, Cardiff & Vale University Health Board, Cardiff, UK; ^e^ School of Medicine, Cardiff University, Cardiff, UK; ^f^ Clinical Educational & Health Psychology, University College London, London, UK; ^g^ School of Medicine, New York University, New York, USA; ^h^ National Center for PTSD, Veterans Affairs Palo Alto Health Care System, Palo Alto, CA, USA

**Keywords:** ICD-11 PTSD, ICD-11 Complex PTSD, Disturbances in Self-Organization (DSO), item response theory, TEPT de la CIE-11, TEPT-C de la CIE-11, perturbaciones en la autoorganización (DSO), teoría de respuesta al ítem., ICD-11 PTSD，ICD-11 复杂 CPTSD ，自我组织失调(DSO)，项目反应理论, • Two ‘sibling disorders’ have been proposed for the 11th version of the International Classification of Diseases (ICD-11): Posttraumatic Stress Disorder (PTSD) and Complex PTSD (CPTSD).• The definition of CPTSD includes the six PTSD symptoms but is distinguished from PTSD on the basis of an additional set of symptoms that reflect ‘Disturbances in Self-Organization’ (DSO).• DSO symptoms are defined by three clusters: (1) affective dysregulation (AD), (2) negative self-concept (NSC), and (3) disturbances in relationships (DR).• This study assessed the performance of 16 DSO symptom indicators from the International Trauma Questionnaire.

## Abstract

**Background**: Two ‘sibling disorders’ have been proposed for the 11^th^ version of the International Classification of Diseases (ICD-11): Posttraumatic Stress Disorder (PTSD) and Complex PTSD (CPTSD). To date, no research has attempted to identify the optimal symptom indicators for the ‘Disturbances in Self-Organization’ (DSO) symptom cluster.

**Objective**: The aim of the current study was to assess the psychometric performance of scores of 16 potential DSO symptom indicators from the International Trauma Questionnaire (ITQ). Criteria relating to score variability and their ability to discriminate were employed.

**Method**: Participants (*N* = 1839) were a nationally representative household sample of non-institutionalized adults currently residing in the US. Item scores from the ITQ were examined in relation to basic criteria associated with interpretability, variability, homogeneity, and association with functional impairment. The performance of the DSO symptoms was also assessed using 1- and 2-parameter item response theory (IRT) models.

**Results**: The distribution of responses for all DSO indicators met the criteria associated with interpretability, variability, homogeneity, and association with functional impairment. The 1-parameter graded response model was considered the best model and indicated that each set of indictors performed very similarly.

**Conclusions**: The ITQ contains 16 DSO symptom indicators and they perform well in measuring their respective symptom cluster. There was no evidence that particular indicators were ‘better’ than others, and it was concluded that the indicators are essentially interchangeable.

## Abstract (es)

**Planteamiento:** Se propusieron dos ‘trastornos hermanos’ para la versión 11 de la Clasificación Internacional de Enfermedades (CIE-11): el Trastorno por Estrés Postraumático (TEPT) y el TEPT Complejo (TEPT-C). Hasta la fecha, ninguna investigación ha intentado identificar los indicadores óptimos de síntomas para el conjunto de síntomas ‘perturbaciones en la autoorganización’ (DSO, por sus siglas en inglés).

**Objetivo:** El objetivo del presente estudio fue evaluar el desempeño psicométrico de las puntuaciones de 16 posibles síntomas de síntomas de DSO del Cuestionario Internacional de Trauma (ITQ, por sus siglas en inglés). Se emplearon criterios relacionados con la variabilidad de las puntuaciones y su capacidad discriminante.

**Método:** Los participantes (*N* = 1839) fueron una muestra de hogares representativos a nivel nacional de adultos no institucionalizados que actualmente residen en los Estados Unidos (EE.UU.). Se examinaron las puntuaciones de los ítems de la ITQ en relación con los criterios básicos asociados con la interpretabilidad, la variabilidad, la homogeneidad y la asociación con el deterioro funcional. El rendimiento de los síntomas de DSO también se evaluó utilizando modelos de teoría de respuesta de items (IRT, por sus siglas en inglés) de 1 y 2 parámetros.

**Resultados:** La distribución de respuestas para todos los indicadores DSO cumplió con los criterios asociados con la interpretabilidad, la variabilidad, la homogeneidad y la asociación con el deterioro funcional. El modelo de respuesta graduada de 1 parámetro fue considerado el mejor modelo e indicó que cada conjunto de indicadores funcionaba de manera muy similar.

**Conclusiones:** El ITQ contiene 16 indicadores de síntomas de DSO y tienen un buen rendimiento en la medición de sus respectivos grupos de síntomas. No hubo pruebas de que determinados indicadores fueran ‘mejores’ que otros, y se concluyó que los indicadores son esencialmente intercambiables.

## Abstract (zh)

背景：第11版的《国际疾病分类》（ICD-11）中提出了两个‘兄弟疾病’：创伤后应激障碍（PTSD）和复杂 PTSD（CPTSD）。目前为止没有研究试图识别‘自我组织失调’症状簇的最优症状指标。

目标：本研究目的在于使用《国际创伤问卷》（ITQ）评估16个可能的 DSO 症状指标分数的心理测量学表现，并使用相关指标考察其分数变异性和区分能力。

方法：1839名被试是目前居住在美国（US）的民间（non-institutionalized）成年人的全国代表性家庭样本。考察ITQ 的项目分和可解释性、变异性、同质性有关的基本标准的关联，以及它与功能损害的关联。DSO 症状的表现使用1各或者2个参数的项目反应理论（IRT）模型进行评估。

结果：所有 DSO指标的反应分布都符合和可解释性、变异性、同质性、功能损害有关的标准。1参数反应模型是最佳模型，说明每个指标组都有相似的表现。

结论：ITQ 包含的16个 DSO 症状指标都可以很好测量对应的整治促。没有证据说明某个指标‘更优于’其它。可以总结认为这些指标本质上都是可以互换的。

Two ‘sibling disorders’ have been proposed for the 11^th^ version of the International Classification of Diseases (ICD-11): Posttraumatic Stress Disorder (PTSD) and Complex PTSD (CPTSD) (Maercker et al., 2013). PTSD is defined by three clusters each containing two symptoms (see Brewin, Lanius, Novac, Schnyder, & Galea, 2009; Maercker et al., 2013): (1) re-experiencing of the trauma in the present (Re), (2) avoidance of traumatic reminders (Av), and (3) a persistent sense of threat that is manifested by increased arousal and hypervigilance (Th). In contrast, the definition of CPTSD includes the six PTSD symptoms as well as an additional set of symptoms that reflect ‘Disturbances in Self-Organization’ (DSO). These DSO symptoms are defined by three clusters: (1) affective dysregulation (AD), (2) negative self-concept (NSC), and (3) disturbances in relationships (DR). The DSO symptom clusters are intended to capture the pervasive psychological disturbances that typically arise following exposure to multiple and repeated traumas (e.g. childhood abuse, being a prisoner of war). Selection of symptoms representative of each cluster was guided by findings from research on Disorders of Extreme Stress Not Otherwise Specified (DESNOS), an earlier version of the CPTSD profile, where those selected were symptoms frequently endorsed by patients in the field trials for the fourth edition of the *Diagnostic and Statistical Manual of Mental Disorders* (DSM-IV; American Psychiatric Association [APA], 1994) (van der Kolk, Roth, Pelcovitz, Sunday, & Spinazzola, 2005), and identified as among the most frequent and distressing by clinicians in an expert consensus survey (Cloitre et al., 2011).

**Figure 1.**
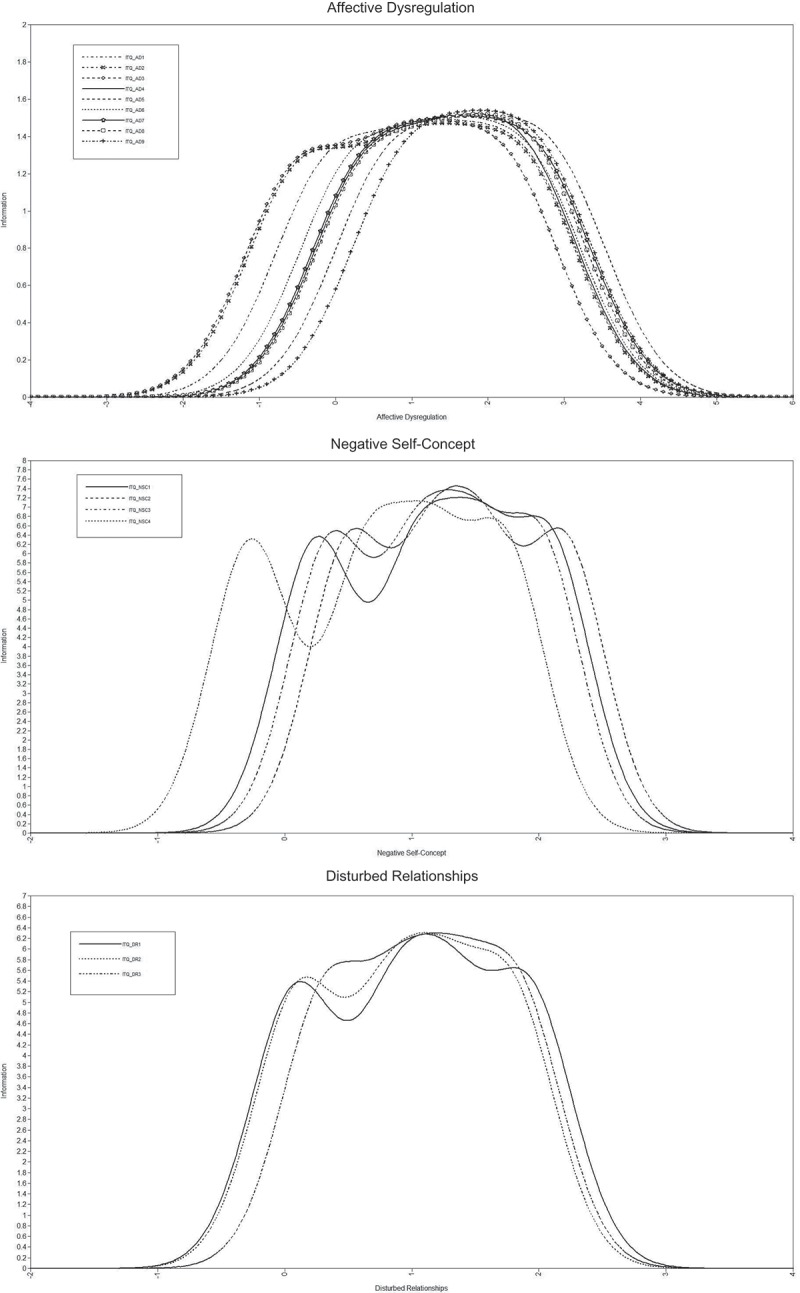
Item information curves for DSO indicators.

ICD-11 guidelines recommend that disorders include a limited but clinically meaning number of symptoms (Reed, 2010). Consistent with these guidelines, the measurement and psychometric assessment of ICD-11 PTSD has limited each cluster to be represented by two symptoms (Brewin et al., 2009). There is an emerging consensus on the specific symptoms that describe and can be used to assess ICD-11 PTSD (Maercker et al., 2013). However, research to reduce the number of symptoms and identify the optimal symptom indicators for the three DSO symptom clusters is still in a preliminary stage. Potential DSO indicators consistent with the ICD-11 characterization of CPTSD have been proposed in the International Trauma Questionnaire (ITQ; Cloitre, Roberts, Bisson, & Brewin, 2015), a self-report measure specifically designed to capture the ICD-11 diagnoses of PTSD and CPTSD. Initial construct validation studies of the ITQ have been promising (e.g. Hyland, Shevlin et al., 2017; Hyland et al., 2017; Karatzias et al., 2016). However, to date, the focus of the psychometric research has been on testing the latent structure of CPTSD, but there has been no attempt to assess how the DSO symptoms perform in a diagnostic capacity.

Accordingly, the overarching goal of this study is to present and apply a systematic approach for assessing the performance of the proposed DSO symptom indicators, as measured by the ITQ. It is critical that the decision regarding which DSO items to retain is informed through a process of rigorous empirical investigation with samples characterized by different traumatic exposures, and from different cultural and national backgrounds. This will help to ensure that the final symptom profile of PTSD and CPTSD will be internationally applicable and highly replicable. Consistent with advances in the formulation of the ICD-11 PTSD assessment, our desire is to identify two well performing items for each DSO cluster. Additionally, our goal is that the AD cluster be represented using one symptom that reflects emotional hyper-activation, and one symptom that reflects emotional hypo-activation, as these were two important aspects of AD identified in an ICD-11 case-controlled field study (Keeley et al., 2016).

The current study assessed the performance of 16 potential DSO symptoms in three linked analytical phases. In Phase 1, the scores of the DSO symptoms were examined to determine if they met basic criteria associated with interpretability, variability, homogeneity, and association with functional impairment. In Phase 2, the performance of the DSO symptoms was assessed using 1- and 2-parameter item response theory (IRT) models. This provided information on how well the indicators measured their respective dimension (discrimination) at levels that would be useful for diagnostic purposes (difficulty). In Phase 3, the diagnostic rates for CPTSD were calculated based on the use of a refined set of DSO symptom indicators.

## 1. Method

### 1.1. Participants

The participants for the current study were a nationally representative household sample of non-institutionalized adults currently residing in the US. Data for this study were collected in March 2017 as part of a larger project assessing the construct validity of the ICD-11 proposals for PTSD and CPTSD. Data were collected using an existing online research panel that is representative of the entire US population. Panel members are randomly recruited through probability-based sampling. Inclusion criteria for the current study were that respondents be aged between 18 and 70 years at the time of the survey, and have experienced at least one traumatic event in their lifetime. A total of 3953 participants were screened to meet the inclusion criteria; a total of 1839 people qualified as valid cases for inclusion in the final analyses (eligibility rate = 46.3%). The survey design oversampled among females and minority populations (African American and Hispanic), each at a 2:1 ratio. To adjust for this oversampling, the data have been weighted to be representative of the entire US adult population. All self-report surveys were completed online (median time of completion = 18 minutes). Individuals received no payment for participation in the survey but were incentivized to participate through entry into a raffle for prizes. Ethical approval for the study was granted by the ethical review board of the institution to which one of the authors is affiliated. The weighted socio-demographic characteristics of the sample are presented in Table 1.

**Table 1.** Weighted sociodemographic characteristics of the sample (*N* = 1839).

	% (*n*)
**Sex**	
Male	48.0 (883)
Female	52.0 (956)
**Age in years**	
18–29	22.0 (405)
30–44	27.7 (510)
45–59	31.2 (573)
60+	19.1 (351)
**Education**	
Less than high school	9.1 (168)
High school	28.7 (528)
Some college	30.3 (558)
Bachelor’s or higher	31.8 (585)
**Race/ethnicity**	
White, Non-Hispanic	63.8 (1173)
Black, Non-Hispanic	11.8 (217)
Other, Non-Hispanic	6.3 (115)
Hispanic	16.9 (310)
2+ Races, Non-Hispanic	1.3 (24)
**Marital status**	
Married	55.3 (1016)
Widowed	2.4 (44)
Divorced	9.0 (166)
Separated	1.9 (36)
Never married	23.3 (428)
Living with a partner	8.1 (149)
**Region**	
Northeast	18.1 (333)
Midwest	20.9 (385)
South	38.2 (702)
West	22.8 (420)
**Employment status**	
Employed	71.1 (1307)
Not employed	28.9 (532)
**Income, US$**	
0–19,999	10.8 (199)
20,000–34,999	11.0 (202)
35,000–74,999	29.8 (547)
75,000 or more	48.5 (891)

### 1.2. Measures

#### 1.2.1. ICD-11 PTSD and CPTSD

The ITQ (Cloitre et al., 2015) is a development-stage self-report measure of ICD-11 PTSD and CPTSD symptoms. The ITQ initially assesses an index trauma and, with this traumatic event in mind, respondents are instructed to indicate how much they have been bothered by six PTSD symptoms in the past month using a five-point Likert scale ranging from ‘Not at all’ (0) to ‘Extremely’ (4). There are three items that screen for functional impairment associated with the PTSD symptoms: ratings of the degree of impairment in (1) relationships and social life, (2) work or ability to work, and (3) other important aspects of life such as parenting, school/college work or other important activities. The internal reliability (Cronbach’s alpha) of the six PTSD items used for diagnostic purposes was satisfactory (α = .89), as were the reliabilities for the Re (α = .80), Av (α = .89), and Th (α = .80) clusters.

For the 16 DSO symptoms, participants are asked to respond to a set of questions reflecting how they typically feel, think about themselves, and relate to others. Nine items capture the AD cluster, five measuring hyper-activation (AD1-AD5) and four measuring hypo-activation (AD6-AD9). Four items capture the NSC cluster (NSC1-NSC4), and three items capture the DR cluster (DR1-DR3) (see Table 2 for all items). There are three items that screen for functional impairment associated with the DSO symptoms: ratings of the degree of impairment in (1) relationships and social life, (2) work or ability to work, and (3) other important aspects of life such as parenting, school/college work or other important activities. All questions are answered using a five-point Likert scale ranging from ‘Not at all’ (0) to ‘Extremely’ (4). The internal reliability of the 16 DSO items was satisfactory (α = .94), as were the reliability estimates for the AD (α = .88), NSC (α = .93), and DR (α = .91) clusters.

**Table 2.** Item content and response frequencies for DSO indicators.

	Scale value							
Item Content	0 Not at all (%)	1 A little bit (%)	2 Moderately (%)	3 Quite a bit (%)	4 Extremely (%)	% Missing	Correlations	
							Item-total	FI
**Affective Dysregulation**								
AD1. I react intensely to things that don’t seem to affect other people so much	47.4	28.3	14.2	7.4	2.8	.6	.61	.45*
AD2. When I am upset, it takes me a long time to calm down.	36.9	35.1	16.9	8.3	2.9	.9	.61	.44*
AD3. My feelings tend to be easily hurt.	35.7	34.4	15.7	9.9	4.3	.8	.60	.44*
AD4. I experience episodes of uncontrollable anger.	62.6	21.8	9.2	3.7	2.7	1.1	.61	.44*
AD5. I do things that people have told me are dangerous or reckless	70.0	17.4	7.9	3.2	1.5	1.2	.50	.38*
AD6. I feel numb or emotionally shut down.	56.8	24.5	9.8	6.3	2.5	1.0	.70	.56*
AD7. I am the kind of person who has difficulty experiencing feelings of pleasure or joy	61.7	21.6	9.6	5.1	2.0	1.5	.70	.58*
AD8. When I am under stress or confronted with reminders of my trauma, I often feel that the world is distant or that the world seems different	63.3	22.1	8.7	3.7	2.2	1.6	.68	.54*
AD9. When I am under stress or confronted with reminders of my trauma, I often feel outside my body or feel that there is something strange about my body.	75.6	13.3	6.2	3.0	1.9	2.0	.63	.57*
**Negative Self-Concept**								
NSC1. I feel like a failure.	59.3	24.8	8.3	5.0	2.5	.9	.76	.64*
NSC2. I feel worthless.	68.4	18.0	6.2	5.4	1.9	1.3	.77	.66*
NSC3. I often feel ashamed of myself whether it makes sense or not.	63.3	20.5	7.8	5.4	3.0	1.2	.80	.68*
NSC4. I feel guilty about things I have done or failed to do.	40.2	33.5	12.5	8.6	5.3	1.3	.74	.59*
**Disturbed Relationships**								
DR1. I feel distant or cut off from people.	53.5	26.4	8.5	7.9	3.8	1.3	.80	.67*
DR2. I find it hard to stay emotionally close to people.	54.5	24.0	9.3	7.2	5.0	2.0	.76	.65*
DR3. I avoid relationships because they end up being too difficult or painful.	62.2	18.2	9.0	6.1	4.5	2.0	.71	.62*

FI = Sum of Functional Impairment items; * *p* < .05.

### 1.3. Analytic strategy

The current study contained three linked analytical phases. In Phase 1, the 16 DSO symptoms were examined to determine item performance and to identify any potentially problematic indicators. The performance of the items was assessed according to four a priori criteria. These criteria were originally proposed by Clarke and Watson (1995) to ensure that: (1) the maximal amount of item level information is retained, (2) attenuated correlations were avoided, and (3) the measure has the ability to discriminate at different points on the underlying continuum. These criteria have been formalized by Lamping et al. (2002) who proposed explicit cut-off values; these cut-off values are helpful in evaluating item performance, but we apply them descriptively rather than prescriptively, as the appropriateness of the values may differ depending on the nature of the instrument being developed. Criterion 1 related to interpretability; problematic interpretability was indicated by missing data of ≥ 10.0% for a given indicator. Criterion 2 related to the variability of responses for each indicator; potential floor and/or ceiling effects were indicated by ≥ 70.0% of responses in one category, while restricted range was indicated by one or more categories with zero responses. Criterion 3 related to item homogeneity; items with an item-total correlation ≥ .30 were deemed to be satisfactory. Criterion 4 related to the relationship between each DSO symptom and levels of functional impairment; adequate associations with functional impairment was indicated by positive correlations ≥ .30.

In Phase 2, a series of increasingly restrictive multi-dimensional IRT models were specified and tested to find the best-fitting and most parsimonious model. These were based on the Graded Response Model (GRM) for polytomous items (Samejima, 1969) as it accommodates ordered response categories. From this model the discrimination (a) and difficulty (b) parameters were estimated for all DSO indicators. The discrimination parameter is the probit regression that relates the latent variable, theta *θ* (with a mean of 0 and a variance of 1), to the normally distributed response variable (y*) that is assumed to underlie the observed responses; higher values indicate increased discriminatory power and provide more information. For each indicator four difficulty parameters (b_1_, b_2_, b_3_, b_4_) are estimated that represent ‘cut-points’ on the underlying trait (*θ*). The GRM is based on cumulative category boundaries; for example, threshold b_1_ represents the level of *θ* where an individual has a probability of .50 of endorsing 0 (‘Not at all’) compared to all higher categories (e.g. 0 vs 1, 2, 3, 4). Similarly, b_2_ is the level of *θ* where an individual has a probability of .50 of endorsing 0 (‘Not at all’) or 1 (‘A little bit’) compared to all higher categories (e.g. 0, 1 vs 2, 3, 4). Each model included three correlated latent variables (AD, NSC, DR) with their respective indicators loading only on one latent variable. A 2-parameter GRM was initially specified where the discrimination and difficulty parameters were estimated for all indicators. Subsequently, a 1-parameter model was specified where the item discrimination parameters were constrained to be equal for items loading on each latent variable. This is ‘within cluster equality’ where the discrimination parameters for the AD, NSC, and DR cluster were constrained equal, but differences across clusters was permitted. The difficulty parameters were unconstrained for all models. Two baseline models were also specified and tested in order to help evaluate the fit of the other models; a single-factor 2-parameter model and a single-factor 1-parameter model. A well-fitting model would also indicate that the assumption of local independence has not been violated.

In Phase 3, two symptom indicators were selected to represent each DSO cluster (AD, NSC, DR) based on the findings from Phases 1 and 2. The fit of two empirically supported factorial models of CPTSD were assessed using confirmatory factor analysis (CFA). These models were: (1) a first-order, correlated, six-factor model in which two items are used to measure each PTSD (Re, Av, Th) and DSO (AD, NSC, DR) cluster; and (2) a second-order, correlated, two-factor model where the covariations between Re, Av, and Th are explained by a second-order ‘PTSD’ factor, and the covariations between AD, NSC, and DR are explained by a second-order ‘DSO’ factor. Additionally, prevalence rates for CPTSD were estimated based on the refined set of DSO symptom indicators. Following from the specification of ICD-11 PTSD (Maercker et al., 2013), the diagnostic criteria for PTSD requires that one of two symptoms be present for the Re, Av, and Th clusters, along with endorsement of one of three indicators of functional impairment associated with these symptoms. Similarly, following ICD-11 characterization of CPTSD (Maercker et al., 2013), the formulation of the diagnostic criteria requires that the PTSD criteria be met; that one of two symptoms be present from the AD, NSC, and DR clusters; along with endorsement of one of three indicators of functional impairment associated with these symptoms. For all symptoms and measures of functional impairment, endorsement was indicated by a score of ≥ 2 (‘Moderately’) on the Likert response scale.

All IRT and CFA models were estimated using Mplus 7.1 (Muthén & Muthén, 2013) using robust weighted least squares estimator (WLSMV) with a probit link based on the polychoric correlation matrix of latent continuous response variables. Goodness of fit for each model was assessed with a range of fit indices including the chi-square (*χ^2^*), the comparative fit index (CFI; Bentler, 1990), and the Tucker-Lewis Index (TLI; Tucker & Lewis, 1973). A non-significant *χ^2^* and values greater than .90 for the CFI and TLI were considered to reflect acceptable model fit. Additionally, the Root Mean Square Error of Approximation (RMSEA; Steiger, 1990) was reported, where a value < .05 indicated close fit and values up to .08 indicated reasonable errors of approximation (Jöreskog & Sörbom, 1993).

## 2. Results

The most commonly reported worst (index) traumas were ‘Sudden death of a loved one as an adult’ (26.0%), ‘Transportation accident as an adult’ (11.1%), ‘Sudden death of a loved one as a child’ (8.8%), and ‘Serious illness or injury as an adult’ (5.7%).

### 2.1. Phase 1

The distribution of responses for all DSO indicators are presented in Table 2. The scores for all indicators were positively skewed with the lower response categories being the most frequently endorsed. No indicators had a large amount of missing data (all ≤ 2.0%) or restricted range (no empty response categories). Two indicators from the AD cluster (AD5: *I do things that people have told me are dangerous or reckless*; and AD9: *When I am under stress or confronted with reminders of my trauma, I often feel outside my body or feel that there is something strange about my body*) had 70.0% or more of the responses in the ‘Not at all’ category. Homogeneity was satisfactory with all item-total correlations > .30. All indicators correlated positively, significantly, and > .30 with levels of functional impairment.

Overall, these results suggest that the majority of the DSO symptoms perform well with respect to the four a priori criteria described above. Each symptom possesses satisfactory interpretability, homogeneity, and associations with functional impairment; and, with only minor exceptions for AD5 and AD9, the DSO symptoms possess satisfactory variability.

### 2.2. Phase 2

All 16 DSO symptoms were used in the IRT models. The fit statistics for the baseline model, 2-parameter model, and 1-parameter model with within cluster equality constraints are reported in Table 3. Although the chi-square statistics were statistically significant for all models this should not lead to their rejection, as the power of the chi-square is positively related to sample size and tends to reject models based on large sample sizes (Tanaka, 1987). The RMSEA showed that the 1-factor 1-parameter and 2-parameter baseline models did not fit the data. The RMSEA, CFI, and TLI indicated acceptable model fit for both the 1-parameter model with within cluster equality constraints on the discrimination parameters and the 2-parameter GRM. Cheng and Rensvold (2002) suggested that the difference in CFIs is a reliable index for assessing model constraints, with a difference > .01 indicating a ‘significant’ difference. The difference between the CFIs for the 1-parameter and 2-paramter model was .003, suggesting that the models do not differ meaningfully. Overall, the 1-parameter model with within cluster equality constraints was considered the best model as it is more parsimonious than the 2-parameter model and the fit of the two models does not differ significantly. The parameter estimates from this model are reported in Table 4.

**Table 3.** Fit statistics for the graded response IRT models of the 16 DSO indicators.

Model	Chi-square (*df*) *p*	RMSEA (90% CI)	CFI	TLI
Baseline 1: one-factor 1 parameter	4452.930 (119) .00	.141 (.137–.145)	.906	.906
Baseline 2: one-factor 2 parameter	2337.706 (104) .00	.108 (.104–.112)	.952	.944
1. 2-parameter GRM	1260.258 (101) .00	.079 (.075–.083)	.975	.970
2. 1-parameter GRM (within cluster equality)	1433.614 (114) .00	.079 (.076–.083)	.972	.970

GRM = graded response model; *df* = degrees of freedom; CFI = Comparative Fit Index; TLI = Tucker Lewis Index; RMSEA = Root-Mean-Square Error of Approximation.

**Table 4.** Graded response model item parameter estimates for DSO indicators.

	Item Parameters (se)				
	Discrimination	Difficulty (Thresholds)			
Item	A	b_1_	b_2_	b_3_	b_4_
**Affective Dysregulation**					
AD1. I react intensely to things that don’t seem to affect other people so much	1.214 (.033)	−0.103 (.056)	1.093 (.060)	2.000 (.071)	3.008 (.104)
AD2. When I am upset, it takes me a long time to calm down.	1.214 (.033)	−0.528 (.057)	0.914 (.059)	1.915 (.070)	2.987 (.100)
AD3. My feelings tend to be easily hurt.	1.214 (.033)	−0.577 (.058)	0.829 (.058)	1.682 (.066)	2.696 (.084)
AD4. I experience episodes of uncontrollable anger.	1.214 (.033)	0.504 (.059)	1.591 (.069)	2.391 (.085)	3.033 (.122)
AD5. I do things that people have told me are dangerous or reckless	1.214 (.033)	0.827 (.064)	1.808 (.078)	2.651 (.094)	3.472 (.145)
AD6. I feel numb or emotionally shut down.	1.214 (.033)	0.271 (.058)	1.400 (.068)	2.126 (.084)	3.083 (.110)
AD7. I am the kind of person who has difficulty experiencing feelings of pleasure or joy	1.214 (.033)	0.468 (.059)	1.521 (.069)	2.317 (.083)	3.239 (.129)
AD8. When I am under stress or confronted with reminders of my trauma, I often feel that the world is distant or that the world seems different	1.214 (.033)	0.536 (.059)	1.660 (.068)	2.463 (.077)	3.170 (.104)
AD9. When I am under stress or confronted with reminders of my trauma, I often feel outside my body or feel that there is something strange about my body.	1.214 (.033)	1.091 (.062)	1.925 (.071)	2.608 (.086)	3.272 (.113)
**Negative Self-Concept**					
NSC1. I feel like a failure.	2.765 (.093)	0.693 (.114)	2.944 (.130)	4.228 (.135)	5.757 (.186)
NSC2. I feel worthless.	2.765 (.093)	1.412 (.124)	3.235 (.137)	4.267 (.142)	6.074 (.203)
NSC3. I often feel ashamed of myself whether it makes sense or not.	2.765 (.093)	1.001 (.117)	2.904 (.132)	4.057 (.147)	5.540 (.171)
NSC4. I feel guilty about things I have done or failed to do.	2.765 (.093)	−0.734 (.106)	1.856 (.124)	3.197 (.143)	4.760 (.176)
**Disturbed Relationships**					
DR1. I feel distant or cut off from people.	2.531 (.093)	0.236 (.100)	2.276 (.117)	3.247 (.126)	4.834 (.154)
DR2. I find it hard to stay emotionally close to people.	2.531 (.093)	0.305 (.101)	2.146 (.120)	3.163 (.128)	4.471 (.148)
DR3. I avoid relationships because they end up being too difficult or painful.	2.531 (.093)	0.845 (.106)	2.324 (.122)	3.387 (.134)	4.608 (.157)

The correlations between the factors were all positive and statistically significant (AD & NSC, *r* = .79; AD & DR, *r* = .81; DR & NSC, *r* = .82). The discrimination parameters (a) for all items were statistically significant, but the indicators for the AD dimension were lower than those for NSC and DR. The threshold parameters indicate that there is variability in the ‘difficulty’ of the items. For example, for NSC4 (*I feel guilty about things I have done or failed to do*) the first threshold is -.734 whereas it is 1.412 for NSC2 (*I feel worthless*). This indicates that a person’s level on the underlying trait (Negative Self-Concept) needs to be higher in order to endorse the second response category (1, ‘A little bit’) of NSC2 compared to NSC4.

The item characteristics were summarized using item information curves (IICs) for each symptom cluster. Item information is analogous to reliability and indicates the precision of measurement across the underlying trait, however precision is not assumed to be constant for all levels of the underlying trait. An item provides most information where the IICs peaks. The IICs for each symptom cluster are shown in Figure 1.

The IICs for the nine AD items all peak between trait levels of 1.00 and 2.50. These are desirable properties for items that are to be used to discriminate at the upper end of the continuum for diagnostic purposes. AD2 (*When I am upset, it takes me a long time to calm down*) and AD3 (*My feelings tend to be easily hurt*) are good indicators for providing information between −0.50 and 2.00 on the underlying trait, whereas AD5 (*I do things that people have told me are dangerous or reckless*) and AD9 (*When I am under stress or confronted with reminders of my trauma, I often feel outside my body or feel that there is something strange about my body*) are better at providing information at the upper end of the trait between 1.50 and 3.00. Three of the four NSC indicators provide most information between .50 and 2.50 on the underlying trait, with NSC4 (*I feel guilty about things I have done or failed to do*) performing better at the lower end of the trait (albeit providing information between .50 and 2.00 on the underlying trait). The three DR indicators all perform very similarly providing most information between .50 and 2.00 on the underlying trait.

Overall, the indicators within each cluster have similar characteristics. Within each cluster the indicators had the same discrimination, and the variability in difficulty parameters was limited. From this, it can be concluded that the indicators from each cluster are largely interchangeable, or ‘tau-equivalent’, with all indicators providing most information above the mean of the underlying trait.

### 2.3. Phase 3

It was not possible to clearly select a specific set of indicators per DSO cluster based on the empirical evidence derived from Phase 1 and Phase 2. Rather, results indicated that all items within each cluster functioned relatively similarly. As such, it was decided to randomly select five different DSO symptom sets (two indicators per cluster were randomly selected); for each dataset, the CFA models were fitted and diagnostic rates were calculated. One theoretically informed constraint was placed on the item selection process: we ensured that for every model the AD factor included one item from the hyper-activation set (AD1–5) and one item from the hypo-activation set (AD6–9). The resulting five randomly generated symptom sets, their model fit results, and their corresponding diagnostic rates are reported in Table 5.

**Table 5.** Model fit statistics for CPTSD based on five randomly generated DSO symptom sets.

	*χ2*	*df*	*p*	CFI	TLI	RMSEA (90% CI)	Dx %
**Six-factor correlated model**							
Model 1 (AD3 & 6, NSC1 & 4, DR1 & 2)	159	39	.000	.995	.992	.041 (.034–.048)	4.3
Model 2 (AD1 & 6, NSC1 & 2, DR1 & 2)	211	39	.000	.995	.991	.049 (.043–.056)	3.9
Model 3 (AD5 & 7, NSC3 & 4, DR2 & 3)	124	39	.000	.996	.993	.035 (.028–.042)	4.1
Model 4 (AD4 & 9, NSC2 & 3, DR1 & 2)	158	39	.000	.995	.992	.041 (.034–.048)	3.8
Model 5 (AD2 & 8, NSC1 & 3, DR1 & 3)	159	39	.000	.995	.991	.041 (.035–.048)	3.8
**Two-factor second-order model**							
Model 1 (AD3 & 6, NSC1 & 4, DR1 & 2)	172	47	.000	.995	.993	.038 (.032–.044)	4.3
Model 2 (AD1 & 6, NSC1 & 2, DR1 & 2)	214	47	.000	.995	.993	.044 (.038–.050)	3.9
Model 3 (AD5 & 7, NSC3 & 4, DR2 & 3)	143	47	.000	.995	.994	.033 (.027–.040)	4.1
Model 4 (AD4 & 9, NSC2 & 3, DR1 & 2)	308	47	.000	.990	.985	.055 (.049–.061)	3.8
Model 5 (AD2 & 8, NSC1 & 3, DR1 & 3)	347	47	.000	.987	.982	.059 (.053–.065)	3.8

Estimator = WLSMV; *N* = 1834; *χ2** = ***Chi-square Goodness of Fit statistic; *df* = degrees of freedom; *p* = Statistical significance; CFI = Comparative Fit Index; TLI = Tucker Lewis Index; RMSEA (90% CI) = Root-Mean-Square Error of Approximation with 90% confidence intervals; Kappa values range from .86 to .92. For second-order model PTSD-DSO factor correlations ranged from .71 to .77 and all statistically significant (*p* < .05).

For each randomly generated symptom set, the fit statistics for the correlated six-factor model, and the two-factor, second-order, model of CPTSD were excellent. The CFI, TLI, and RMSEA values each indicated that the proposed models provided close fit to the sample data. The diagnostic rates of CPTSD were highly consistent across the five symptom set variations, with estimates ranging from 3.8 to 4.3%. Moreover, Cohen’s Kappa values ranged from .86 to .92 indicating a very high level of overlap in the individuals receiving a diagnosis of CPTSD across the five symptom sets.

Overall, results from Phase 3 suggested that a random selection of any two indicators for each DSO cluster (with one hyper-activation and one hypo-activation item used to reflect the AD factor) produced excellent model fit and consistent diagnostic estimates. These results provide further support for the conclusion that the indicators within the AD, NSC, and DR clusters are largely interchangeable.

## 3. Discussion

Following the narrative guidelines set forth for ICD-11 PTSD and CPTSD by the WHO’s Department of Mental Health and Substance Abuse (First, Reed, Hyman, & Saxena, 2015), members of the ICD-11 Working Group for Disorders Specifically Associated with Stress developed the ITQ (Cloitre et al., 2015) as a standardized method of measuring the specific symptom content of these diagnoses. Following the work of Brewin et al. (2009), and aligned with the goal of ICD-11 to maximize clinical utility (First et al., 2015), a six-symptom model of PTSD has been proposed and widely validated (e.g. Forbes et al., 2015; Hansen, Hyland, Armour, Elklit, & Shevlin, 2015; Hyland, Brewin, & Maercker, 2017; Tay, Rees, Chen, Kareth, & Silove, 2015). Consequently, the ICD-11 model is more parsimonious compared to the model of PTSD proposed by the DSM-5 (APA, 2013). For example, there are 27 combinations of symptoms that can produce a diagnosis of PTSD in ICD-11, while there are 636,120 combinations of symptoms that can produce a diagnosis of PTSD in DSM-5 (Galatzer-Levy & Bryant, 2013). The development of the ITQ incorporated these six core PTSD symptoms.

Although there is consensus regarding (a) the structure of ICD-11 CPTSD (PTSD plus DSO symptoms) and (b) that the DSO dimension shall be described in terms of three correlated symptoms clusters (AD, NSC, DR), there has yet to be a clearly defined proposal regarding the exact number of symptoms that should be used to measure each cluster, and what those symptoms should be. For the purposes of the ITQ development, a number of potential symptom indictors were generated for each DSO cluster based on the DSM-IV field trials for CPTSD (van der Kolk et al., 2005) and expert clinical feedback (Cloitre et al., 2011). This resulted in the development of nine AD symptoms, reflecting hyper-activation and hypo-activation experiences, four NSC symptoms, and three DR symptoms. Early factorial validity studies that utilized the full set of potential symptom indicators provided support for the CPTSD proposals (Hyland, Shevlin et al., 2017; Hyland et al., 2017; Karatzias et al., 2016). Despite this empirical support there was a need to reduce the number of DSO symptoms in the ITQ to ensure that the CPTSD diagnosis aligns with the ICD-11’s emphasis on clinical utility and use of as few symptom indicators without compromising validity and diagnostic utility (Maercker et al., 2013). Corresponding to the proposals for PTSD in ICD-11, we argue that the identification of two symptoms per DSO cluster would be advantageous, however the challenge is to identify six DSO symptoms that function effectively across diverse trauma populations which vary in terms of type of traumatic exposure, nationality, cultural background, demographics, and community or clinical status. Additionally, these six DSO symptoms should generate similar (or superior) empirical support for the construct validity of CPTSD as has been observed when employing the full set of symptom indicators. The overarching goal of the current study was to develop a methodological framework that researchers from around the world could replicate to aid in the streamlining of the ITQ. The primary objective of this study was to determine how the application of this methodological approach functioned among a nationally representative sample of the US adult population. It is critical to stress that we did not seek to definitively identify which two items should be selected from the ITQ to measure each DSO construct; rather, we believe it is critical that an evidence base accumulates from multiple independent sources, and that the resulting body of evidence be used to determine the most effective symptom indicators of the AD, NSC, and DR clusters.

The results from Phase 1 of our analysis showed that the scores of the DSO indicators all met the basic criteria associated with (1) interpretability (no evidence of excessive missing data), (2) variability (no evidence of problematic floor or ceiling effects, however AD5 and AD9 did have slightly more responses in the ‘Not at All’ category than would be desirable), (3) item homogeneity (all item-total correlations were robust), and (4) association with functional impairment (all items were adequately associated with functional impairment scores). The IRT results in Phase 2 showed that all AD, NSC, and DR symptoms performed equally well at identifying individuals at different levels of the underlying latent variable (equality of discrimination), however, there was variation in terms of where one needs to score on the underlying latent variable in order to endorse a particular symptom (variability in item difficulty). Although there was some variation in the difficulty of endorsing the DSO symptoms, the item information curves showed that all indicators were providing maximum information at the upper end of the underlying continua, above the mean, which is desirable for diagnostic purposes. Phase 3 results showed that the latent structure of the PTSD and DSO indicators were stable irrespective of the indicators that were chosen from each cluster. Moreover, the latent symptom structure models that distinguish between PTSD and DSO symptomatology, in line with ICD-11 proposals, and previously supported using the full set of DSO items, were found to provide very close fit to the data. Indeed, the overall fit of these models based on the refined symptom sets were superior to those that have been previously reported (Hyland, Shevlin et al., 2017; Karatzias et al., 2016). It is important that psychometric models not only provide good model fit, but that they also possess clinical utility (Shevlin, Hyland, Karatzias, Roberts, & Bisson, 2017). Our findings showed that irrespective of which set of six DSO symptom indicators were selected, the CPTSD prevalence rates remained highly consistent. Together, these findings suggest that the scores from the DSO symptoms, as measured by the ITQ, all have acceptable psychometric properties, operate in a similar way, generate extremely good model fit, and consistently identify the same individuals meeting diagnostic criteria for CPTSD.

The results of the current study should be interpreted cautiously. This study simply represents the first effort to streamline the number of DSO symptoms to be used to model CPTSD, and to investigate the performance of the CPTSD diagnosis using a refined set of DSO symptom indicators. It should be stressed that the results of this study were based on a nationally representative sample of US adults and, as such, indicates how well the DSO indictors perform when the primary aim is to identify the presence of CPTSD. This sample is less likely to be exposed to multiple and repeated traumas that are considered to differentiate PTSD and CPTSD. Indeed, the most commonly reported index traumas in this sample (death of a loved one as a child/adult, transportation accident, serious illness) are not those that have been proposed to be important in the development of CPTSD, such as childhood sexual abuse. In a clinical sample, there is likely to be participants who have experienced multiple forms of childhood trauma and maltreatment with or without additional adult traumatization (see Karatzias et al., 2016). The indicators that can successfully discriminate between different levels of severity of DSO may be different from those that can identify probable ‘cases’ in the population. Furthermore, certain items may function differently depending upon the culture, nationality, or language of the respondent. It is our belief that that the development of a robust body of empirical evidence drawn from internationally diverse trauma samples, in combination with clinical feedback and clinical interpretation, will lead to the most effective selection of DSO indicators and, thus, a description of CPTSD that maximizes clinical, and research, utility.

Overall this study represents only one stage in the selection of appropriate DSO indicators. The development of the DSO clusters used in this analysis have a research and clinical heritage. Brewin et al. (2017) describes how the current DSO clusters share similarity with the previous ICD-10 diagnosis F62.0 ‘Enduring personality change after catastrophic experience’ and ‘Disorders of Extreme Stress Not Otherwise Specified’ (DESNOS) which was included in the Appendix to DSM-IV and were ‘derived largely from review of the empirical literature’ (p. 3). There is also evidence that the proposed DSO clusters represent those symptoms that clinicians reported to be the most appropriate. Cloitre et al. (2011) reported the findings from a survey of 50 expert clinicians who had been asked to rate 11 CPTSD symptom domains in terms of frequency and associated impairment; the AD, NSC, and DR clusters were reported to be the most frequently observed and endorsed as substantial contributors to impairment. However, the fact that the DSO clusters have a strong basis in research and clinical relevance does not mean that symptom selection should be based exclusively on psychometric analysis. Future research needs to include (1) continued assessment of the clinical relevance of the DSO clusters and specific indicators, (2) the identification and assessment of ‘gold-standard’ criterion variables for each of the clusters, (3) further exploration of the association between DSO clusters and different forms of functional impairment and disability, and (4) provide service users with further opportunities to contribute to the development of diagnostic criteria (see d’Ardenne & Heke, 2014).

In conclusion, this study showed that each of the DSO symptom indicators from the ITQ were acceptable measures of the respective symptom cluster. Further research using participants who have experienced specific traumas, or polytrauma, and other cultural groups is required.

## Supplementary Material

Supplementary material
