# Supplementary material for: A psychometric assessment of Disturbances in Self-Organization symptom indicators for ICD-11 Complex PTSD using the International Trauma Questionnaire
Source: Eur J Psychotraumatol. 2018 Jan 17;9(1):1419749. doi: 10.1080/20008198.2017.1419749 (PMC5774393; doi:10.1080/20008198.2017.1419749)
Supplement: Supplementary material [file ZEPT_A_1419749_SM2441.pptx]

## Slide 1
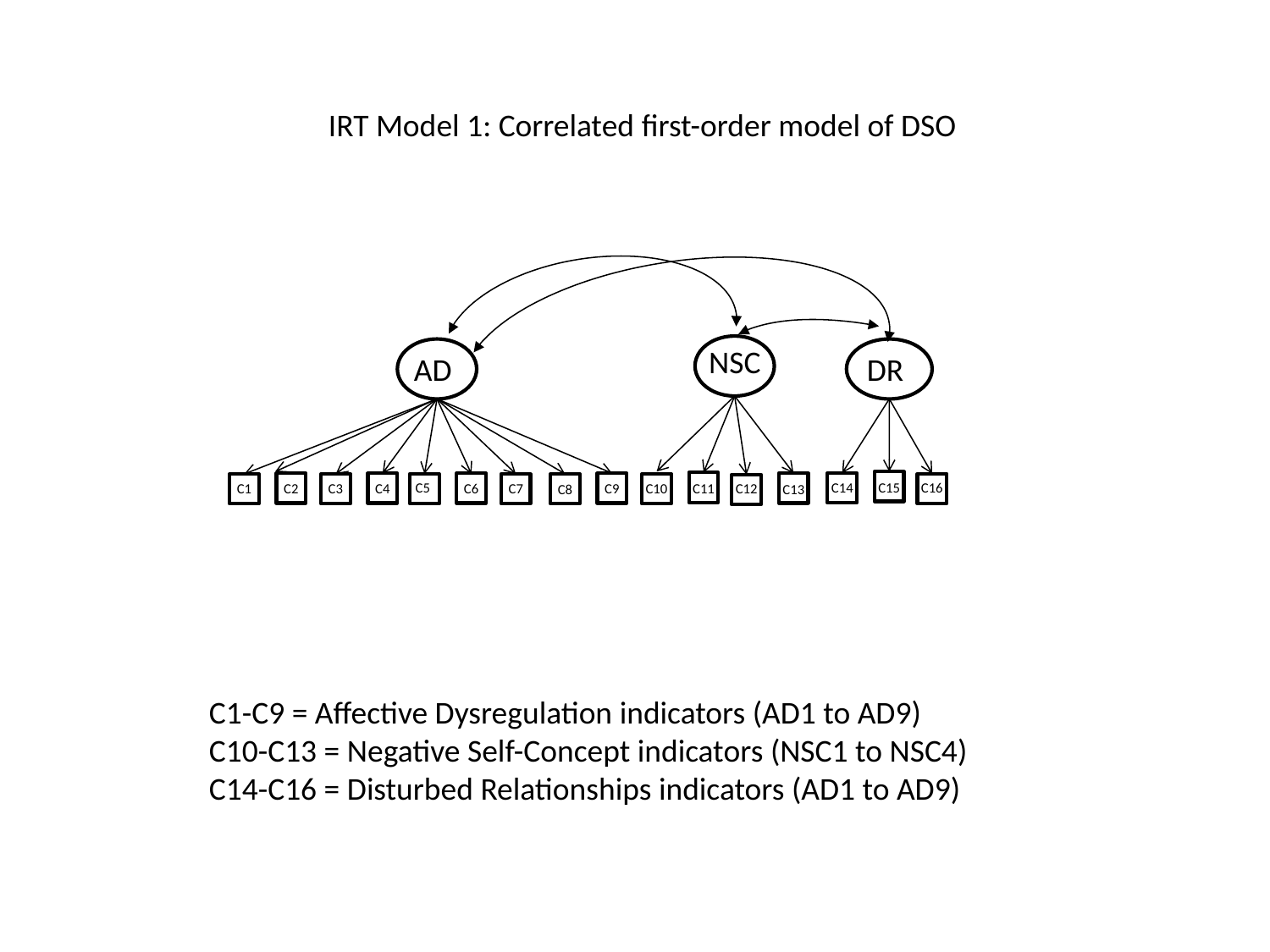

IRT Model 1: Correlated first-order model of DSO
NSC
AD
DR
C14
C15
C5
C16
C3
C7
C10
C1
C11
C12
C2
C4
C6
C9
C8
C13
C1-C9 = Affective Dysregulation indicators (AD1 to AD9)
C10-C13 = Negative Self-Concept indicators (NSC1 to NSC4)
C14-C16 = Disturbed Relationships indicators (AD1 to AD9)

## Slide 2
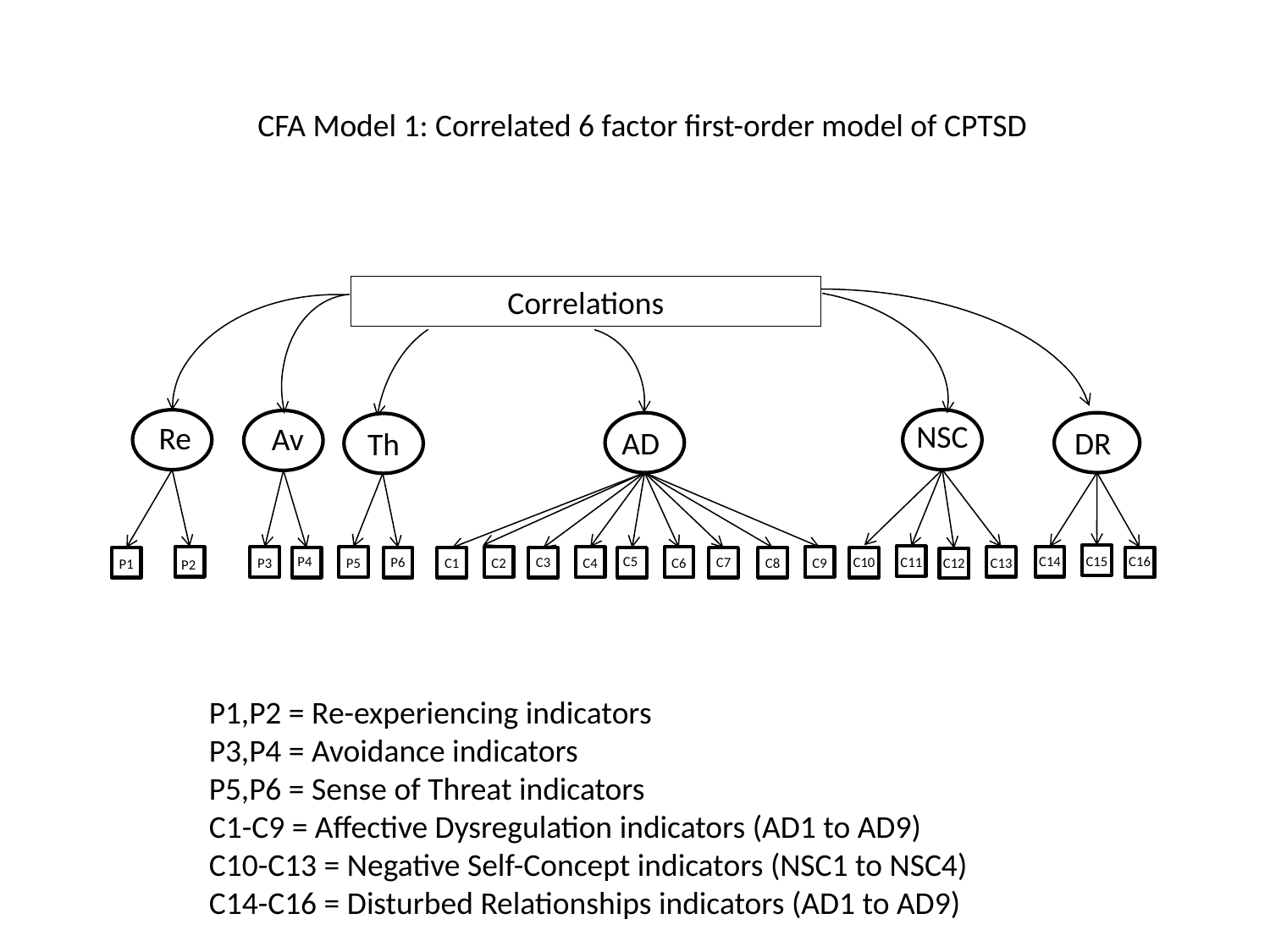

CFA Model 1: Correlated 6 factor first-order model of CPTSD
Correlations
NSC
Re
Av
AD
DR
Th
C14
C15
P4
C5
C16
P6
C3
C7
C10
C11
C12
P3
P5
C2
C4
C6
C9
C1
C8
C13
P1
P2
P1,P2 = Re-experiencing indicators
P3,P4 = Avoidance indicators
P5,P6 = Sense of Threat indicators
C1-C9 = Affective Dysregulation indicators (AD1 to AD9)
C10-C13 = Negative Self-Concept indicators (NSC1 to NSC4)
C14-C16 = Disturbed Relationships indicators (AD1 to AD9)

## Slide 3
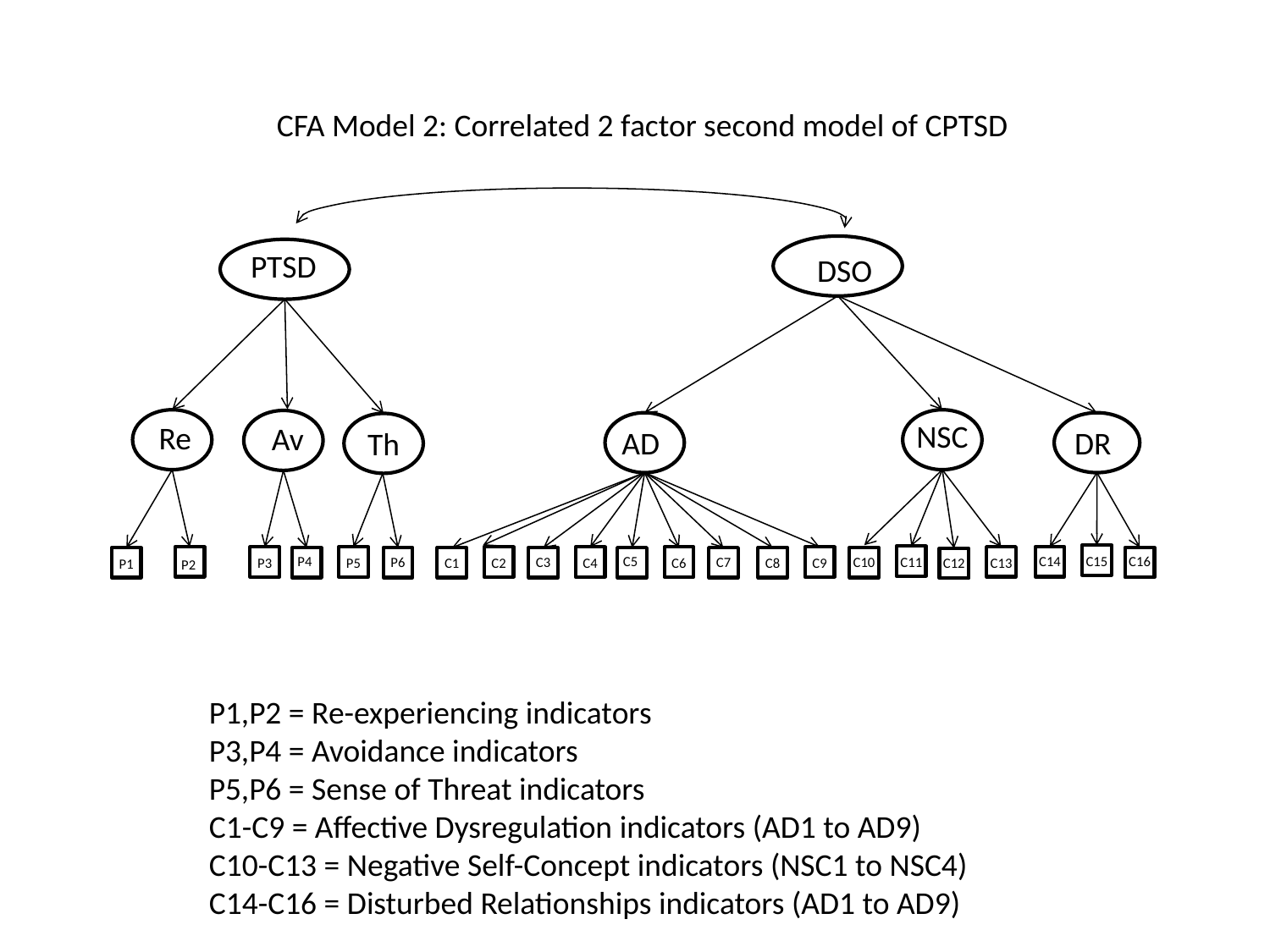

CFA Model 2: Correlated 2 factor second model of CPTSD
PTSD
DSO
NSC
Re
Av
AD
DR
Th
C14
C15
P4
C5
C16
P6
C3
C7
C10
C11
C12
P3
P5
C2
C4
C6
C9
C1
C8
C13
P1
P2
P1,P2 = Re-experiencing indicators
P3,P4 = Avoidance indicators
P5,P6 = Sense of Threat indicators
C1-C9 = Affective Dysregulation indicators (AD1 to AD9)
C10-C13 = Negative Self-Concept indicators (NSC1 to NSC4)
C14-C16 = Disturbed Relationships indicators (AD1 to AD9)
